# 

**Published:** 1898-09-17

**Authors:** 


					THE hospital. " The standard of highest purity."-^.
CADBURY'S TOT 1 ^ OADBU^^
ABSOLUTELY PURE
COCOA
COOOA " i JM& NO DRUGS OR ALKALIES USED.
>? -^-c^htrtiLAScofv Westfrn Infirmary
No. 625. Vol. XXIV. Saturday,
Sept. 17th, 1898.
[Subscription Tern*.] pRIC;E TWOPENCE.

				

